# Nutritional properties and biological activities of kiwifruit (*Actinidia*) and kiwifruit products under simulated gastrointestinal *in vitro* digestion

**DOI:** 10.29219/fnr.v63.1674

**Published:** 2019-04-08

**Authors:** Tingting Ma, Tian Lan, Tonghui Geng, Yanlun Ju, Guo Cheng, Zhiluo Que, Guitian Gao, Yulin Fang, Xiangyu Sun

**Affiliations:** 1 Key Laboratory of Agro-Products Processing, Key Laboratory of Agro-products Quality and Safety Control in Storage and Transport Process, Ministry of Agriculture and Rural Affairs/Institute of Food Science and Technology, Chinese Academy of Agricultural Sciences, Beijing, China; 2 College of Enology, College of Food Science and Engineering, Heyang Viti-viniculture Station, Northwest A&F University, Yangling, China; 3 College of Food Engineering and Nutritional Science, Shaanxi Normal University, Xi’an, China

**Keywords:** kiwifruit, kiwifruit products, nutritional properties, in vitro digestion, digestive characteristics, antioxidant capacity

## Abstract

**Background:**

Kiwifruit is one of the most commercialized fruits on the international market, which has notable high nutritional and medicinal value with many health benefits. In addition to being consumed fresh, numerous kiwifruit products are popular, such as kiwifruit juice, vinegar, dried slices, jam, wine, yogurt, and jelly. Although many studies have described the nutritional properties of kiwifruit, investigations on the nutritional properties of kiwifruit products remain limited, especially for kiwifruit products made from raw kiwifruit.

**Methods:**

Nutritional properties and biological activities of kiwifruit and kiwifruit products, as well as the digestive and absorption characteristics of their nutritional substances, were investigated.

**Results:**

Kiwifruit, juice, wine, and vinegar were observed to be rich in vitamin C (VC) and polyphenol and exhibited high biological activities, whereas dried kiwifruit slices and jam showed higher amounts of mineral elements. During oral digestion, VC and polyphenol showed similar absorption characteristics, while mineral elements exhibited a number of different trends. A good release rate of all nutritional substances was observed during stomach digestion, while the release rate decreased in serum-available, colon-available, and post-colonic fractions. Eating dried slices and jam supplied high amounts of mineral elements, while eating kiwifruit supplied the most comprehensive nutritional substances. The biological activities detected in raw foodstuffs were much higher than those detected after *in vitro* digestion. Furthermore, kiwifruit and wine showed the highest biological activities, while dried kiwifruit slices showed the lowest biological activities.

**Conclusion:**

These results increased our understanding of the nutritional properties of kiwifruit and its products, providing new information and scientific recommendations to consumers for kiwifruit consumption and to producers for kiwifruit production.

## Popular scientific summary

- Kiwifruit, juice, wine and vinegar were rich in VC and polyphenol.
- Dried slices and jam supplied high amounts of mineral elements.
- A good release rate of all nutritional substances was observed during stomach digestion.
- Biological activities in raw foodstuff were much higher than after in vitro digestion.
- Eating kiwifruit supplied the most comprehensive nutritional substances.

## 

Kiwifruit (*Actinidia*) is one of the most commercialized fruits on the international market (1). However, it is native to China and was replanted in New Zealand in approximately 1904, from where it became one of the newest fruit crops to gain international commercial importance (2), and was then replanted back to China in the 1970s. After its development over several decades, China is now the largest producer of kiwifruit in the world (3), with an annual production of approximately 1.06 million tons (accounting for 38% of the total global production) and a planting area of approximately 180,000 hectares (accounting for 59% of the total global planting area) (1). In addition to being consumed fresh, numerous kiwifruit products are popular in China, such as kiwifruit juice, vinegar, dried slices, jam, wine, yogurt, and jelly, especially in Shaanxi province, which is the largest kiwifruit producing area in China (accounting for approximately 61% of the total kiwifruit production in China).

Kiwifruit has notable high nutritional and medicinal value with many health benefits (4), including its laxative activity (5), antidiabetic properties (6), anti-inflammatory properties (7), cardiovascular protective properties (8), and antimicrobial activities against human pathogens (9). These effects are primarily due to the bioactive components present in kiwifruit. The most well-known bioactive compound in kiwifruit is vitamin C (VC) (10), which in some varieties can be as high as 420 mg/100 g kiwifruit, much higher than in other fruits (11). In fact, kiwifruit is considered to be the ‘king of VC’ by the Chinese consumers. In addition, kiwifruit is also rich in polyphenols (3), which have also been reported as potential health-promoting molecules and display a broad spectrum of effects associated with their antioxidant activity (11). Furthermore, kiwifruit is also a good source of mineral element for humans (3, 12), such as Fe, Zn, Cu, Se, and Cr. In addition to these bioactive compounds, the use of the plant growth regulator forchlorfenuron in the cultivation of kiwi trees has caught the attention of consumers in China over the past few years (13). The long-term, excessive, and large-scale use of forchlorfenuron as a plant growth-promoting agent in China (from approximately 1995 to 2004) has had serious consequences and eroded consumer confidence in Chinese kiwifruit, resulting in decreases in Chinese kiwifruit prices (3).

Processing significantly influences the nutritional composition of food (14–17). Although many studies have described the nutritional properties of kiwifruit (1, 3, 4, 10, 11), investigations on the nutritional properties of kiwifruit products remain limited, especially for kiwifruit products made from raw kiwifruit. The health-related effects of bioactive components depend not only on their concentration in foods and the amount consumed, but also on their bioavailability (18). The bioactive components that are abundant in foods are not necessarily those that are present at the highest concentrations in tissues or exhibit biological effects, owing to considerable differences in their bioavailability (19). In recent years, an increasing number of studies have used *in vitro* digestion models as low-cost and high-throughput tools to investigate the gastrointestinal (GI) behavior of foods. These models allow for the fast and inexpensive screening of a large sample set, whereas *in vivo* studies, such as human nutritional studies, are time-consuming, costly, and restricted by ethical concerns (14, 15, 18, 19).

Therefore, the aims of this study were to compare the nutritional properties of kiwifruit and kiwifruit products, including VC, polyphenols, and mineral elements, as well as antioxidant capacity. Furthermore, the forchlorfenuron in kiwifruit was also investigated. A simulated GI *in vitro* digestion model was used to analyze the digestive and absorption characteristics of the nutritional components of kiwifruit and kiwifruit products. The results of this study will increase the understanding of the nutritional properties of kiwifruit and its products, providing new information and scientific recommendations to both consumers and producers of kiwifruit.

## Materials and Methods

### Samples and chemicals

The *Actinidia deliciosa* (*A. deliciosa*) kiwifruits (Hayward) used in this study were donated by Shaanxi Bairui Kiwifruit Research Co. Ltd. in 2017, which were harvested from Zhouzhi county, Shaanxi province, China. All of the kiwifruit materials were at their commercial maturity and were of uniform size. Five popular kiwifruit products in China were made using the above-mentioned kiwifruit materials, including kiwifruit juice (1), vinegar (20), dried slices (21), jam, and wine (22), in our laboratory based on previous reports, with slight modifications or traditional methods. A minimum of 10 kg of kiwifruit was used for the production of each test food to minimize the influence of raw material heterogeneity (14). All kiwifruit and kiwifruit product samples were stored at −20°C for further analysis. The solvents used in this study were of analytical or high-performance liquid chromatography (HPLC) grade.

### In vitro *GI digestion*

The *in vitro* GI digestion assay was performed according to a previously described procedure (18, 19), with slight modifications. Four sequential digestion steps, including mouth (oral), stomach (gastric), small intestine (duodenal), and colon digestions, were included in this model to mimic the *in vivo* GI digestion. For each digestion step, the sample groups were independent such that the mouth step had 12 parallel sets for each food type, while there were nine sets in the stomach step, six sets in the small intestine step, and three sets in the colon step.

#### Mouth digestion

For kiwifruit and dried kiwifruit slices, after being thawed to ambient temperature, kiwifruits were cut into 5-mm cubic pieces to mimic the coarse consistency obtained by chewing (14). Other liquid/fluid kiwifruit products were thawed and used in the digestion model without further pretreatment. For each of the test foods, a 10-g sample was added to an amber glass flask containing 10 mL of an artificial saliva solution (50 mM NaCl, 10 mM NaH_2_PO_4_, and 40 mM NaHCO_3_), after which the pH was adjusted to 6.7–6.9 using 1 M NaOH, followed by the addition of 100 μL of a fresh α-amylase preparation containing 25 U. Then the mixtures were shaken at 100 *g* for 1 min in a shaking incubator at 37°C to simulate agitation in the mouth (14, 23). After 2 h, three samples for each treatment were immediately snap-frozen in liquid nitrogen to stop the reaction, and the samples were stored until further treatment.

#### Stomach digestion

After the mouth digestion, the samples from the mouth phase were mixed with 20 mL of simulated gastric fluid (2 g of NaCl and 450 U pepsin/mL, adjusted pH 1.2 with HCl and brought to a volume of 1 L with distilled water) (24). The mixtures were adjusted to a pH of 1.2, transferred into tubes wrapped in aluminum foil, and then incubated in a water bath at 37°C using an incubated shaker with continuous shaking at 100 *g* for 2 h to simulate gastric digestion. The volume of each sample group was made consistent by adding physiological saline. During processing, the pH of the reaction system was monitored and maintained by adding 1 M HCl using an automatic titration unit (Metrohm, Riverview, FL, USA). After 2 h, three sample groups for each treatment were immediately snap-frozen in liquid nitrogen immediately to stop the reaction, and the samples were stored until further treatment.

#### Small intestine digestion

After the stomach digestion, the remaining sample was placed in a glass beaker and 10 mL of simulated intestinal fluid (7 g of NaH_2_PO_4_, 4 mg/mL pancreatin, and 25 mg/mL bile salts, adjusted to pH 7.5 with NaOH and brought to a volume of 1 L with distilled water) (24) was added. The volume of each sample group was made consistent by adding physiological saline, and NaOH was used to adjust the pH to 7.5. Next, for each sample, a fully filled, bubble-free, and closed dialysis bag (molecular weight cut-off of 12 kDa) containing sufficient NaHCO_3_ at pH 7.5 was placed into a glass beaker containing the sample, which was then sealed with parafilm. The glass beaker was placed in a 37°C water bath in the dark in an incubated shaker with continuous shaking at 100 *g* for 2 h to simulate the small intestine digestion. The pH of the reaction system was monitored and maintained by titrating a 0.25 M sodium hydroxide solution into the reaction vessel using an automatic titration unit (Metrohm, Riverview, FL, USA). Next, the dialysate, that is, the solution contained inside the dialysis bag (the fraction passing through the dialysis membrane), was separated and stored, representing the fraction available for absorption into the circulatory system by passive diffusion (serum-available fraction). In addition, the solution contained outside the dialysis bag, that is, the non-dialyzable fraction, was separated and stored, representing the material that remained in the GI tract and would reach the colon (colon-available fraction). After 2 h, the serum-available fraction and colon-available fraction of three sample tubes for each treatment were collected and acidified with formic acid to pH 2 to neutralize NaHCO_3_, and the samples were then stored until further treatment.

#### Colonic digestion

The fecal inoculums were obtained from fresh feces collected from the entire large intestines of male SD rats (70-day-old animals, average 300 g) immediately after euthanasia (25). A fecal pool of five animals was made, which was immediately homogenized with the culture medium at a ratio of 1:10 (w/v). The fermentation medium was prepared according to a previously described method (26), which was sterilized at 121°C for 30 min before use. An amount of 10 mL of the solution contained outside the dialysis bag from the small intestine digestion was thoroughly mixed with 45 mL of fecal inoculum and 45 mL of fermentation medium using a vortex mixer. The pH of the mixture was adjusted to 8.0 by the addition of 2 M NaHCO_3_. The experimental samples were transferred into different sealed anaerobic tubes in an anaerobic incubator, which were subsequently incubated at 37°C and shaken at 100 *g* for 18 h. Next, the fermentation products were collected and incubated in ice water for 5 min to stop fermentation. Subsequently, the fermentation products were centrifuged at 8,000 *g* for 15 min to separate the supernatants for further analysis.

#### Sample preparation for analyses

Aliquots arising from oral, stomach, intestine (serum-available fraction and colon-available fraction), and colonic digestions were centrifuged at 13,000 *g* for 10 min. All samples were immediately filtered through 0.45-μm pore filters, fractionated in microtubes, and stored at −80°C until analysis.

### VC analysis

The VC content was determined using HPLC method based on CNS GB 5009.86-2016 (27).

### Elemental analysis

Heavy metal elements (Mn, Cd, Hg, and As), macro-elements (N, K, Mg, P, Ca, and Na), and trace elements (Fe, Zn, and Se) were detected using an inductively coupled plasma optical emission spectrometer (ICP-OES, Optima 7000DV, Perkin Elmer, Waltham, Massachusetts, USA) after liquid ashing (4 mL HNO_3_ + 1 mL H_2_O_2_) of the samples in a microwave digestion system (3).

### Determination of the polyphenol contents

Total phenolics (TP), total flavonoids (TFA), total flavan-3-ols (TFO), and total anthocyanins (TA) determinations

The TP content was determined according to the Folin–Ciocalteu colorimetric method (11) and the results were expressed as milligrams of gallic acid equivalents (GAE)/100 g fresh weight (FW) (mg GAE/100 g FW). The TFA content was determined according to a previously described protocol (28) and the results were expressed as milligrams of catechin equivalents (CTE)/100 g FW (mg CTE/100 g FW). The TFO content was estimated using the slightly modified DMACA method (28) and the results were expressed as mg CTE/100 g FW. The TA content was estimated using the pH differential method (29) and the results were expressed as milligrams of cyanidin-3-glucoside (CGE)/100 g FW (mg CGE/100 g FW). All spectrophotometric measurements were performed on a UV-Vis double beam Hitachi U-3010 spectrometer (Hitachi, Kyoto, Japan).

#### Phenolic compound analysis

Sixteen phenolic compound standards were assayed: three flavonols (quercetin, rutin, and phlorizin), four flavan-3-ols [(+)-catechin, L-epicatechin, (−)-epigallocatechin gallate, and (−)-epicatechin gallate], four hydroxycinnamic acids (caffeic acid, ferulic acid, chlorogenic acid, and p-coumaric acid), four hydroxybenzoic acids (vanillic acid, gallic acid, gentisic acid, and salicylic acid), and one stilbene (trans-resveratrol). A Waters Breeze liquid chromatograph equipped with a 1,525 Bin pump, a 2,487 DAD Detector (Waters Corp., Milford, MA, USA), an Autoscience AT-130 column heater, and a Waters XBridge^TM^ Shield RP18 (4.6 mm × 250 mm, 3.5 μm) was used for HPLC analysis. The mobile phase comprised (A), 98% (v/v) acetonitrile containing 2% (v/v) glacial acetic acid, and (B), 2% (v/v) glacial acetic acid. The elution gradient was 5 to 15% A for 60 min, 15% A for 5 min, from 15 to 20% for 1 min, 20% A for 7 min, from 20 to 30% for 1 min, 30% A for 6 min, from 30 to 40% for 1 min, 40% A for 12 min, and from 40 to 5% A for 2 min, with a flow rate at 0.8 mL/min. The injection volume used was 20 μL, and the detection wavelength was 280 nm. The column temperature was set at 30°C. We observed good separation of the 16 phenolic acids under the chromatographic conditions, with R^2^ values of greater than 0.9992, indicating that the 11 standards showed good linear relationships in the concentration ranges tested.

### Antioxidant capacity analysis

Four different methods, namely, 1,1-diphenyl-2-picrylhydrazyl assay (DPPH), 2, 2’-azino-bis (3-ethylbenzothiazoline-6-sulfonic acid) assay (ABTS), oxygen radical absorbance capacity (ORAC), and fluorescence recovery after photobleaching (FRAP), were used in this study. The DPPH scavenging activity and ABTS assays were based on previously described methods (29), with slight modifications. The ORAC and FRAP assays were performed essentially as has been previously described (3), with some modifications. The results are expressed as μM Trolox/100 g FW.

### Evaluation of the inhibitory effects of α-amylase and α-glucosidase

The methods used for the α-amylase enzymatic inhibition assays performed in this study were adapted from those developed in a previous study (30), with slight modifications. In addition, the α-glucosidase inhibitory activity was measured as described in a previous report (30), with slight modifications. The inhibitory activity (I) was calculated using the following equation:

I (%) = [1 – (A_sample_ – A_background_)/A_control_] × 100

### Determination of forchlorfenuron contents

The presence of forchlorfenuron was assayed using our previously described HPLC method (3).

### Bacterial enumeration

After colonic digestion, a previously described method (31) was used to enumerate the number of *Enterobacteriaceae* (using MacConkey selective medium), *Lactobacillus* (using MRS medium agar), *Enterococcus* (using agar with kanamycin, esculin, and sodium azide), and *Bifidobacterium* (using Garch’s medium) bacteria present in the samples. The number of live bacterial cells was assessed using Koch’s plating method.

### Statistical analysis

The experimental results are expressed as means ± standard deviations (SD). Statistical analyses were performed using Data Processing System (DPS, version 7.05, Hangzhou, China).

## Results and discussion

### Changing levels of VC in kiwifruit and kiwifruit products during in vitro GI digestion

**Fig. 1:**
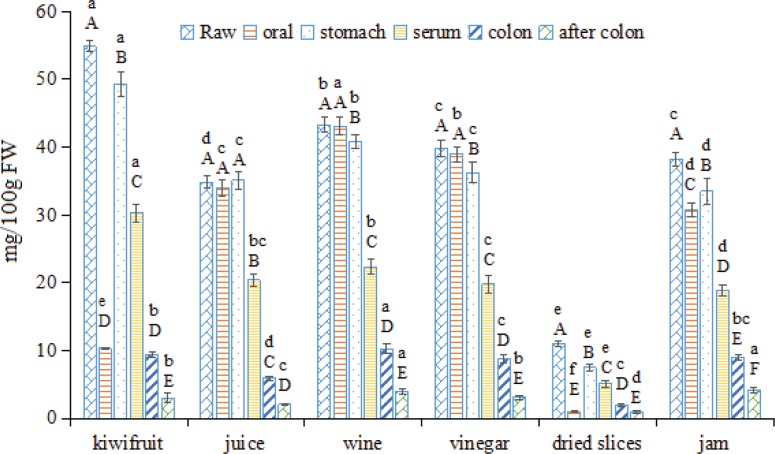
Changes in the VC contents of kiwifruit and kiwifruit products during *in vitro* GI digestion. The different capital letters indicate significant differences among the different digestion phases with same kiwifruit products at *p* < 0.05; the different lowercase letters indicate significant differences among the different kiwifruit products at the same digestion phase at *p* < 0.05.

VC is one of the most notable nutrients of kiwifruit (11) and is much higher in kiwifruit than in any other fruits, such as oranges, strawberries, lemons, and grapefruit (3). In the present study, the raw Hayward kiwifruit had a VC content of 54.86 mg/100 g FW (Fig. 1), which similar to that of a previous report (32). After processing, the VC content decreased in all kiwifruit products, among which wine showed the highest VC content (43.33 mg/100 g FW), followed by vinegar (39.87 mg/100 g FW) and juice (34.82 mg/100 g FW). Dried kiwifruit slices showed the lowest VC content, which was primarily due to the thermal instability of VC, as the processing of dried kiwifruit slices is a heat-intensive process (21). Kiwifruit also showed a higher VC content than dried kiwifruit slices, despite jam production also being a heat-intensive process. This result may have been due to the thermal treatment used to produce dried kiwifruit slices being more intense than that used to make jam.

During oral digestion, the VC content showed an entirely different behavior. Raw kiwifruit showed a much lower VC content, which was only slightly higher than that observed for dried kiwifruit slices, while kiwifruit juice, wine, and vinegar showed similar contents as the raw kiwifruit (with release rates of 97.59, 99.45, and 97.77%, respectively). Furthermore, jam also showed a high release rate (80.38%). This result was primarily due to the difference between solid and liquid/colloid food matrices (19). After the stomach digestion process, the VC content in raw kiwifruit and kiwifruit juice, dried slices, and jam increased, especially in dried slices. This result may be due to the VC being released from the solid dried slices matrix after the intense digestion in the stomach (18). In addition, the VC content decreased slightly in the kiwifruit wine and vinegar samples. This result may have occurred due to VC degradation, as VC was fully released from the liquid wine and vinegar matrix during the oral digestion process, and after the subsequent long-term stomach digestion process, a certain amount of the VC degraded. After stomach digestion, foodstuffs are transported to the small intestine, where specific nutritional substances are then absorbed (serum-available fraction), while others are transferred to the colon (colon-available fraction) (16). In the serum fraction, raw kiwifruit showed the highest VC content (30.29 mg/100 g FW), whereas kiwifruit juice showed the highest VC release rate (58.50%). In contrast, dried kiwifruit slices showed the lowest VC content (5.09 mg/100 g FW) and VC release rate (46.36%), with approximately 30.29, 20.37, 22.38, 19.77, 5.09, and 18.83% of VC absorbed in the small intestine after an intake of 100 g of raw kiwifruit or kiwifruit juice, wine, vinegar, dried slices and jam. Yi et al. (32) Reported that a daily intake of one average-sized kiwifruit (80 g) supplies the required VC (60 mg) in European countries. However, they only considered the VC concentration and not the *in vivo* absorption (bioavailability) of VC; thus, the kiwifruit intake value they obtained might not be so objective. Next, the foodstuffs were transported to the colon, which contains a diverse ecosystem of microorganisms. The VC and other nutrient contents can serve as substrates for the microorganism community in the colon, influencing the microbial ecosystem or continuing on to be absorbed into the serum, which happens with only a small proportion of nutrient contents (33). After colonic fermentation, an average of 5 to 10% of VC remained in the colon, of which dried kiwifruit slices and jam showed the lowest and highest VC contents, respectively, while kiwifruit, juice, wine, and vinegar showed similar VC contents.

### Alteration of mineral elements in kiwifruit and kiwifruit products during in vitro GI digestion

**Fig. 2:**
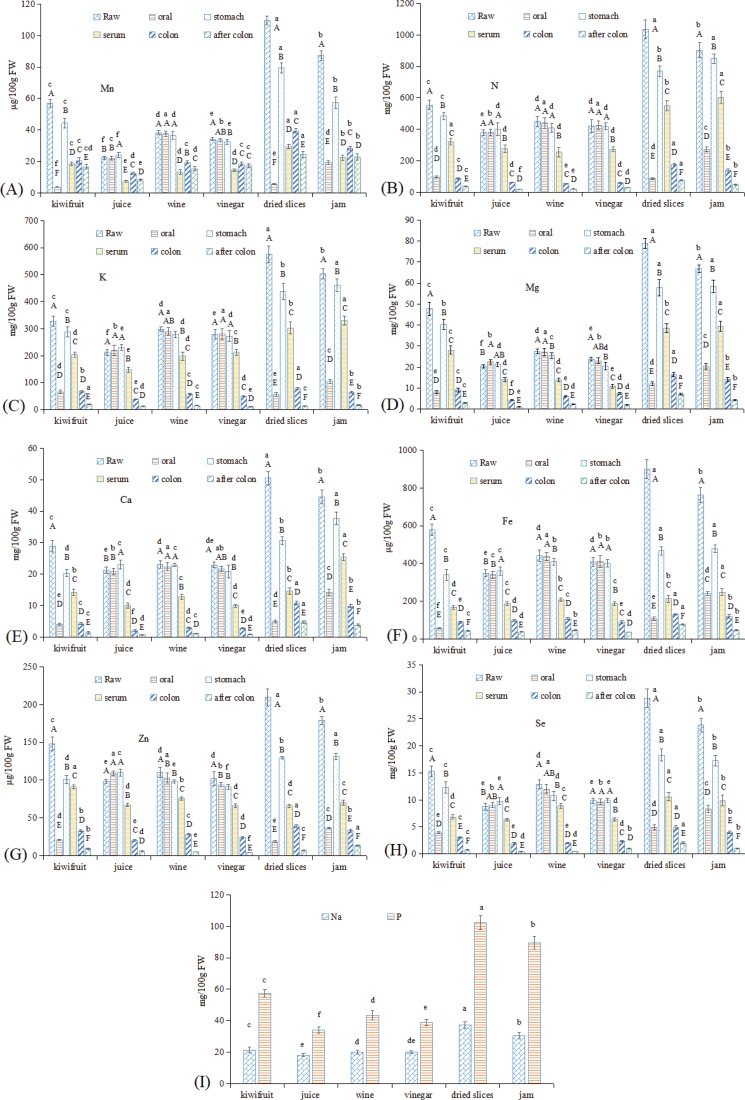
Changes in mineral elements in kiwifruit and kiwifruit products during *in vitro* GI digestion. (A) Mn, (B) N, (C) K, (D) Mg, (E) Ca, (F) Fe, (G) Zn, (H) Se, and (I) Na and P. The different capital letters indicate significant differences among the different kiwifruit items with the same kiwifruit products at *p* < 0.05; the different lowercase letters indicate significant differences among the different kiwifruit products with the same kiwifruit variety at *p* < 0.05.

Kiwifruit is a good source of mineral elements for humans (3, 12). In this study, 13 elements were assayed. As shown in Fig. 2, among the four heavy metal elements assayed, only Mn was present (Fig. 2A), while no Cd, Hg, or As was detected in any of the samples. All six macro-elements (Fig. 2B–E, I) and three trace elements (Fig. 2F–H) were also present in all samples. In contrast to VC, after processing, dried kiwifruit slices showed the highest mineral element contents, followed by jam, which was much higher than observed in raw kiwifruit. This result may be due to the enrichment effect of the processing methods used to make dried kiwifruit slices and jam (21). In addition, kiwifruit juice, wine, and vinegar showed lower mineral element contents than that were observed in raw kiwifruit. The reason for this result may be due to the processing of kiwifruit juice, wine, and vinegar, as a large fraction of element contents were lost with the separation of the pomace (1).

After oral digestion, in raw kiwifruit, Mn showed the lowest release rate (6.77%) among the heavy metal elements (Fig. 2A), followed by the trace element Fe (9.95%) (Fig. 2F), while the release rates of all other elements ranged from approximately 15 to 25%. This result may be due to the different binding forms of elements that resulted in different dissolution rates (34). Kiwifruit juice, wine, and vinegar showed similar Mn contents as raw kiwifruit (with a release rate of approximately 98%), while the release rates observed in kiwifruit jam and dried slices were 22.06 and 5.27%, respectively, close to that observed for raw kiwifruit. The reason for this result may be due to differences between solid and liquid/colloid food matrices (19). After the stomach digestion process, Mn showed a similar increasing trend as VC, with observed release rates ranging from 66 to 107%. Dried kiwifruit slices and jam had higher Mn contents than raw kiwifruit, while kiwifruit juice, wine, and vinegar had lower Mn contents than raw kiwifruit. In the subsequent small intestine digestion, dried kiwifruit slices showed the highest Mn content in the serum fraction, followed by with kiwifruit jam and raw kiwifruit, while kiwifruit juice, wine, and vinegar showed higher Mn release rates than the other three foodstuffs. After colonic fermentation, a certain amount of Mn was still present, with contents ranging from 8.47 to 24.57 mg/100 g FW and release rates ranging from 22.46 to 50.95%.

For the six assayed macro-elements, the digestive characteristics of Na and P were indeterminable (Fig. 2I), primarily due to their concentrations in the blank digestive solutions were too high, which making an evaluation of their bioavailability not possible. It should be noted that these two elements are present in most reagents used for GI simulation (35), and Schulz et al. (34) also reported that the bioavailability of Na was indeterminable in their assays. Among the kiwifruit and kiwifruit products, dried slices showed the highest Na and P contents, followed by jam, while the other three liquid products showed lower contents than that was observed in raw kiwifruit. With respect to N, K, Mg, and Ca, the contents among the kiwifruit and kiwifruit products showed similar trends as N and P (Fig. 2B–E). After oral digestion, raw kiwifruit (approximately 14–20%) and dried kiwifruit slices (approximately 8–15%) showed the lowest release rate of all four elements, while jam showed a higher release rate (approximately 20–30%), and kiwifruit juice, wine, and vinegar showed much higher release rates (approximately 96–109%). After the stomach digestion process, the release rates of raw kiwifruit, dried kiwifruit slices, and jam all greatly increased, approximately 60–70% for dried kiwifruit slices and approximately 80–95% for kiwifruit and jam. In the serum fraction, dried kiwifruit slices and jam showed the highest content of all four elements (except in Ca for dried kiwifruit slices), whereas dried kiwifruit slices showed the lowest release rate, and the release rates for kiwifruit juice, wine, vinegar, and jam were similar. After colonic fermentation, the release rates of the four macro-elements were lower than 10%.

With respect to the three trace elements, the raw kiwifruit and its products exhibited similar trends for Mn and macro-elements (Fig. 2F–H). During the *in vitro* GI digestion, kiwifruit vinegar showed the highest release rate of Fe, and kiwifruit juice showed the highest release rates of Zn and Se, whereas raw kiwifruit and dried kiwifruit slices exhibited the lowest release rates for Fe, Zn, and Se, as was observed in the oral digestion. In addition, the release rates of Fe, Zn, and Se increased for kiwifruit juice after stomach digestion, which may be due to the digestive enzyme promoting the delivery of these three elements (19, 34). In the serum fraction, Fe showed a release rate of approximately 50% in kiwifruit juice, wine, and vinegar, and a release rate of approximately 30% in raw kiwifruit, dried kiwifruit slices, and kiwifruit jam. Zn showed a release rate of approximately 70% in raw kiwifruit, kiwifruit juice, wine, and vinegar, and approximately a 30% release rate in dried kiwifruit slices and kiwifruit jam. For Se, a release rate of approximately 70% was observed in kiwifruit juice, wine, and vinegar, and a release rate of approximately 40% was observed in raw kiwifruit, dried kiwifruit slices, and kiwifruit jam. Typically, the bioavailability of Fe and Zn in most fruits is low, primarily due to low concentrations of protein, which can increase the bioavailability of Fe and Zn by reducing and chelating iron (36). The high release rates of Fe and Zn may be due to the presence of considerable amounts of protein in kiwifruit (3). Schulz et al. (34) also reported a high bioavailability of Fe (approximately 7.6–29.5%) and Zn (approximately 35.8–69.2%) in juçara fruit pulp that had a high protein content. After colonic fermentation, approximately half of these three elements that passed from the colon-available fraction were used by the microorganisms in the colon.

### Alterations of polyphenols in kiwifruit and kiwifruit products during in vitro GI digestion

#### TP, TFO, TFA, and TA

**Fig. 3:**
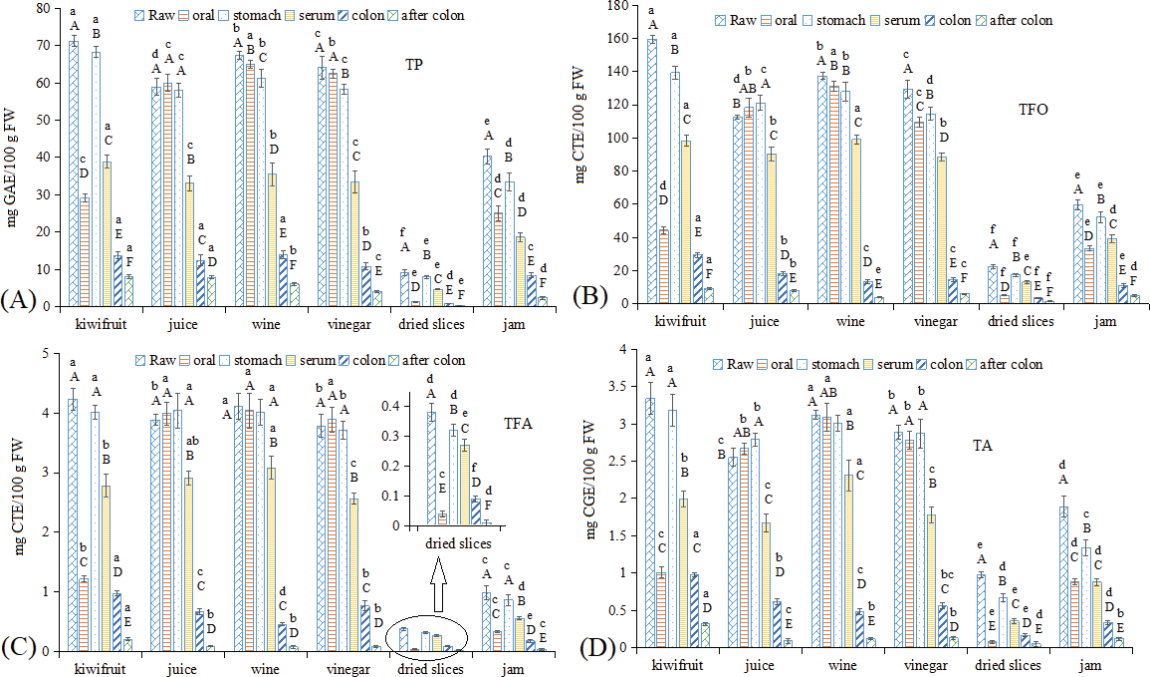
Changes in polyphenol contents in kiwifruit and kiwifruit products during *in vitro* GI digestion. (A) TP, (B) TFO, (C) TFA, and (D) TA. The different capital letters indicate significant differences among the different kiwifruit items with same kiwifruit product forms at *p* < 0.05; the different lowercase letters indicate significant differences among the different kiwifruit products with the same kiwifruit variety at *p* < 0.05.

Many studies have shown that antioxidant phytochemicals (especially polyphenols) consumed via the diet are good resources of natural antioxidants (11, 29). Kiwifruit is also a good source of polyphenols (3). The TP, TFO, TFA, and TA assay results are shown in Fig. 3. Similar to VC, raw kiwifruit showed the highest TP, TFO, TFA, and TA contents, while kiwifruit juice, wine, and vinegar showed slightly lower values than what were observed in raw kiwifruit. Dried kiwifruit slices showed the lowest contents of all four indexes, whereas kiwifruit jam showed higher TP and TA values (56.55 and 56.59% than that of raw kiwifruit, respectively) and had low TFO and TFA values (37.33 and 23.40% than that of raw kiwifruit, respectively). This result was primarily due to the thermal instability of polyphenols, of which TFO and TFA were more unstable (37).

After oral digestion, the solid food matrix kiwifruit and dried kiwifruit slices, as well as the fluid food matrix jam, all showed higher release rates in all four polyphenol indexes than for mineral elements (Fig. 2) and VC (Fig. 1), indicating that polyphenols had the best dissolution among these three nutritional substances. Interestingly, after oral digestion, kiwifruit juice had a higher polyphenol value than what was observed in the raw juice, primarily due to the effects of oral digestive enzymes. Lingua et al. (16) also reported increased TFA and TA contents after the oral digestion of red wines. The release rates of kiwifruit wine and vinegar showed a similarly good release (approximately 86–102%). In the subsequent stomach digestion process, kiwifruit juice, wine, and vinegar showed similar release rates as that after oral digestion, while the release rates of raw kiwifruit, dried kiwifruit slices, and kiwifruit jam increased significantly, with dried kiwifruit slices still exhibiting the lowest value (except that jam had the lowest TP content). In the serum fraction, TFO and TFA showed a better release rate (range 60–70%), followed by TA, while the release rate of TP was lower (approximately 50%). Only 31% of wine TP was found in the serum fraction in a study by Lingua et al. (16), much lower than what was observed in this study. In addition, a 60.55% release rate of TP was observed for blueberry wine by Celep et al. (33), similar to that observed in this study. The difference in these results is primarily due to the different materials used and the different *in vitro* GI digestion systems used (19, 34). The TA release rate in the serum fraction was approximately 40–60%, which is consistent with the report by Lingua et al. (16) but somewhat different from the report by Yang et al. (38), primarily because Yang et al. did not consider the digestive enzymes in their *in vitro* GI digestion model. After colonic fermentation, most of the release rates of the four macro-elements were lower than 10%, especially for the dried kiwifruit slices, which exhibited TP, TFO, TFA, and TA values of 1.21, 6.57, 2.63, and 5.10%, respectively. This result may be due to dietary fiber being enriched in the dried kiwifruit slices compared to the other kiwifruit foodstuffs, as dietary fiber can promote the growth of colonic microorganisms, and more polyphenol compounds were used by the microorganisms in this group than the others assayed.

#### Individual phenolic compounds

**Fig. 4:**
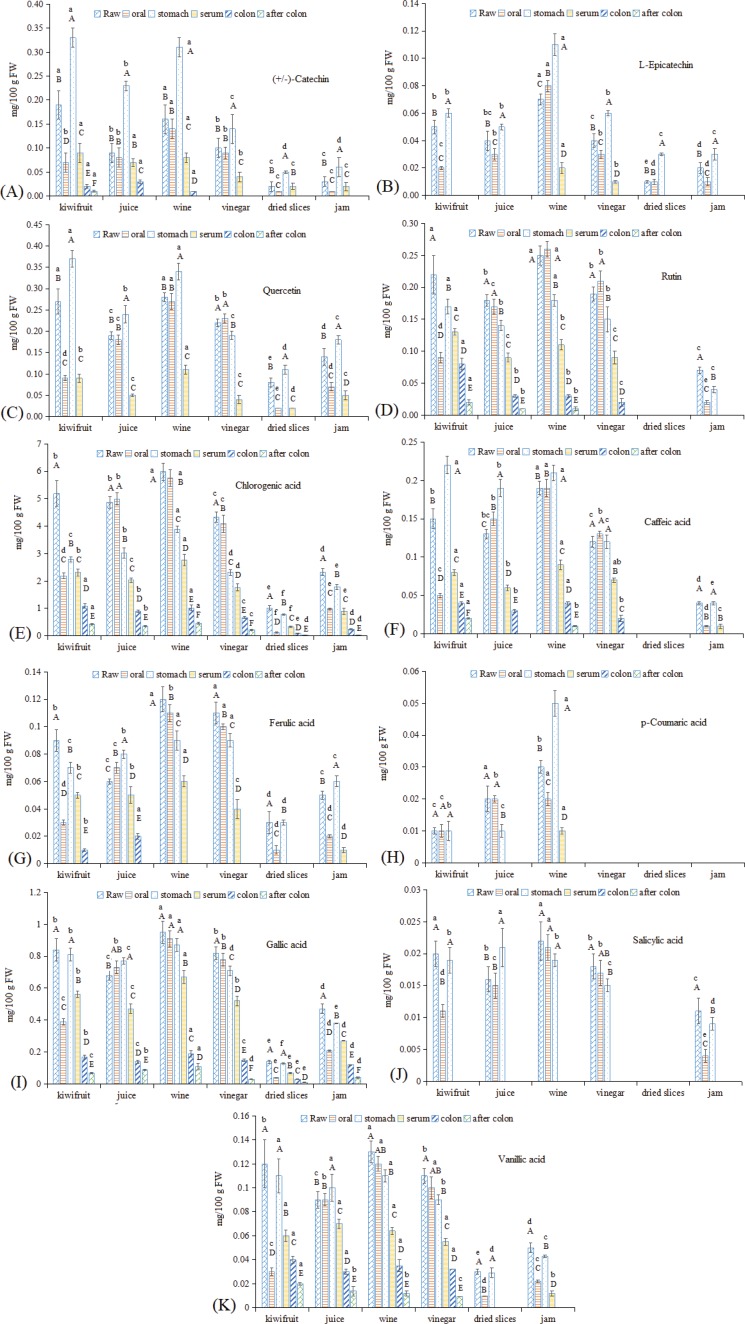
Changes in individual phenolic compounds in kiwifruit and kiwifruit products during *in vitro* GI digestion. (A) (+/−)-catechin, (B) l-epicatechin, (C) quercetin, (D) rutin, (E) chlorogenic acid, (F) caffeic acid, (G) ferulic acid, (H) p-coumaric acid, (I) gallic acid, (J) salicylic acid, and (K) vanillic acid. The different capital letters indicate significant differences among the different kiwifruit varieties with same kiwifruit product forms at *p* < 0.05; the different lowercase letters indicate significant differences among the different kiwifruit products with the same kiwifruit variety at *p* < 0.05.

To gain a better understanding of the changes in polyphenols in kiwifruit and kiwifruit products during *in vitro* GI digestion, the polyphenol composition after *in vitro* GI digestion was investigated by assaying 16 phenolic compounds, the results of which are shown in Fig. 4. Chlorogenic acid, gallic acid, and (+/−)-catechin were the primary phenolic compounds detected, which were consistent with a previous report (3). After thermal processing, the individual phenolic compounds decreased substantially, especially for the dried kiwifruit slices. In fact, no rutin, caffeic acid, p-coumaric acid, or salicylic acid was detected in the dried kiwifruit slices, and the levels of other phenolic compounds were also very low. Similar to the observed TP contents, kiwifruit jam showed higher levels of phenolic compounds than what was observed for the dried kiwifruit slices and lower than the other three liquid kiwifruit stuffs. In addition, kiwifruit wine showed higher levels of phenolic compounds than raw kiwifruit, except (+/−)-catechin. This result may be due to the fermentation process influencing the polyphenol contents via the metabolism of *Saccharomyces cerevisiae* (16, 29).

After oral digestion, the individual phenolic compounds showed similar changing trends with TP, where raw kiwifruit, dried kiwifruit slices, and kiwifruit jam showed low release rates, while kiwifruit juice, wine, and vinegar showed higher release rates (approximately 100%). Lingua et al. (16) reported an increasing trend of individual phenolic compounds in wine after oral digestion, and the observed differences may be due to differences in the *in vitro* GI digestion systems used (19, 34). After the subsequent stomach digestion process, rutin levels decreased significantly in kiwifruit juice, wine, and vinegar (Fig. 4D), whereas quercetin levels significantly increased in all kiwifruit foodstuffs except vinegar (Fig. 4C). This result may be due to rutin being an O-glycoside with a disaccharide sugar moiety (rutinoside, composed of rhamnose and glucose), and it is a well-known fact that glycosidic linkages tend to cleave under acidic pH conditions and/or as a result of enzymatic reactions. Interestingly, because quercetin is the aglycone skeleton of rutin, after the cleavage of the sugar moiety of rutin, the quercetin content increases (Celep et al., 2015) (33). (+/−)-Catechin and l-epicatechin (Fig. 4A, B) also showed increasing trends that were in line with the TFA results (Fig. 3C). Furthermore, the caffeic acid levels also increased (Fig. 4F), which may be due to the occurrence of some unknown metabolism of phenolic compounds during the stomach digestion process. In the serum fraction, ferulic acid, p-coumaric acid, salicylic acid, and l-epicatechin were not detected, primarily because their initial concentrations in raw foodstuffs were too low. For the other phenolic compounds, rutin, caffeic acid, vanillic acid, and (+/−)-catechin showed a release rate of approximately 50–70%, while quercetin and chlorogenic acid showed lower release rates (approximately 30–40%). After colonic fermentation, quercetin, ferulic acid, p-coumaric acid, salicylic acid, vanillic acid, (+/−)-catechin, and l-epicatechin were not detected or were present at very low levels, whereas other phenolic compounds showed release rates of approximately 10%.

### Changes in the antioxidant capacity of kiwifruit and kiwifruit products during in vitro GI digestion

**Fig. 5:**
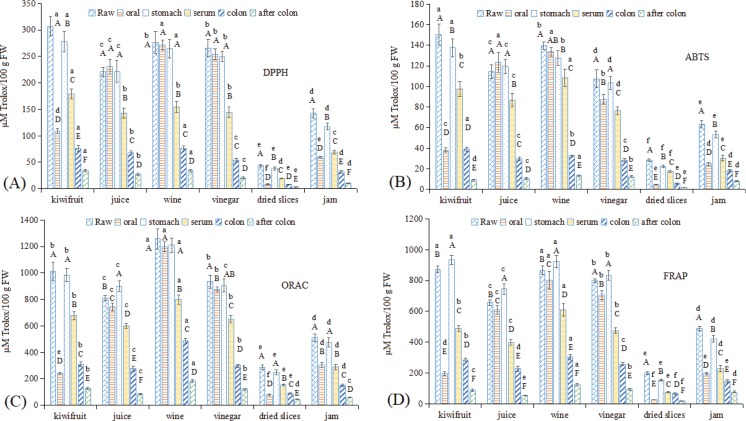
Changes in the antioxidant capacities in kiwifruit and kiwifruit products during *in vitro* GI digestion. (A) DPPH; (B) ABTS; (C) ORAC; (D) FRAP. The different capital letters indicate significant differences among the different kiwifruit varieties with same kiwifruit product forms at *p* < 0.05; the different lowercase letters indicate significant differences among the different kiwifruit products with the same kiwifruit variety at *p* < 0.05.

Fruits have always been considered to have positive effects on human health, primarily because of the antioxidant substances they contain and their associated antioxidant activities (3). Thus, the antioxidant activities in kiwifruit and kiwifruit products during *in vitro* GI digestion were evaluated in this study (Fig. 5), with four methods used based on previous reports (3, 29). Kiwifruit wine showed the highest antioxidant activity by the ORAC method, while raw kiwifruit showed the highest antioxidant activity using the other three methods. This difference occurred due to the differences in the methods (29). In addition, kiwifruit juice, wine, and vinegar showed high antioxidant activity using all four methods, while that of kiwifruit jam was higher than dried kiwifruit slices and was lower than the other three liquid kiwifruit foodstuffs. These results were consistent with the polyphenol and VC results (Figs. 1, 3, 4), and previous reports have also demonstrated a strong correlation between antioxidant capacity due to polyphenols (3, 11, 16, 34) and VC values (3, 11) in kiwifruit and other foodstuffs.

After stomach digestion, the antioxidant capacity showed similar changing trends as polyphenols and VC, where dried kiwifruit slices showed the lowest antioxidant capacity, followed by raw kiwifruit and kiwifruit jam, while kiwifruit juice, wine, and vinegar showed higher antioxidant capacities. In the serum fraction, the antioxidant capacity in all samples decreased, but the observed trend was similar to the stomach digestion. Raw kiwifruit and kiwifruit wine showed the highest antioxidant capacity in serum, while dried kiwifruit slices showed the lowest antioxidant capacity. In the colonic fractions, the antioxidant capacities of samples were approximately 20–30% of that of the original samples, while after colonic fermentation, only approximately 7–15% of the antioxidant capacities remained.

### Changes in the inhibitory effects of α-amylase and α-glucosidase in kiwifruit and kiwifruit products during in vitro GI digestion

**Fig. 6:**
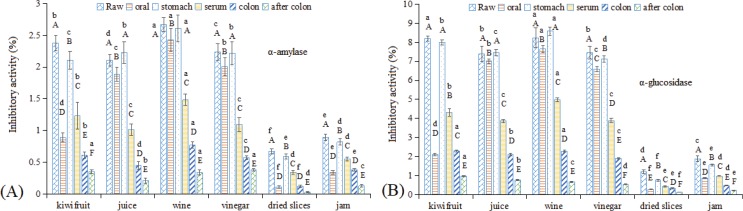
Changes in the inhibitory effects of α-amylase and α-glucosidase in kiwifruit and kiwifruit products during *in vitro* GI digestion. (A) α-amylase and (B) α-glucosidase. The different capital letters indicate significant differences among the different kiwifruit varieties with same kiwifruit product forms at *p* < 0.05; the different lowercase letters indicate significant differences among the different kiwifruit products with the same kiwifruit variety at *p* < 0.05.

Foods, especially fruits rich in polyphenols, offer an attractive means of managing postprandial hyperglycemia for type 2 diabetes by controlling starch breakdown and intestinal glucose absorption (30, 39). Thus, the inhibitory effects of α-amylase and α-glucosidase in kiwifruit and kiwifruit products during *in vitro* GI digestion were investigated (Fig. 6). Raw kiwifruit showed a 2.38% inhibition ratio for α-amylase, while kiwifruit juice, wine, and vinegar showed 2.11, 2.67, and 2.24% α-amylase inhibition ratios, respectively. In contrast, the observed inhibition ratios for dried kiwifruit slices and kiwifruit jam were only 0.67 and 0.89% (Fig. 6A). With respect to α-glucosidase, higher inhibition ratios were obtained than what were observed for α-amylase (e.g. 8.19 and 1.21%, α-glucosidase inhibition ratios were observed for kiwifruit and dried kiwifruit slices) (Fig. 6B). This result was in accordance with observations done by Kwon et al. (39) for wines, where better inhibition ratios were observed for α-glucosidase than for α-amylase. Ma et al. (30) also reported a similar result for polyphenol extracts from *Sphallerocarpus gracilis* stems and leaves. After oral digestion, a similar trend like the one observed for the polyphenol results was observed (Fig. 3), that is, kiwifruit juice, wine, and vinegar showed slightly lower inhibition ratios than what was observed in raw foodstuffs, while raw kiwifruit, dried kiwifruit slices, and kiwifruit jam showed much lower values than in foodstuffs before digestion. Specifically, during stomach digestion, dried kiwifruit slices showed only half the inhibition ratio for α-glucosidase as the original sample. In the serum-available fraction, an approximately 47–60% inhibition ratio for α-amylase and α-glucosidase was observed, except for dried slices for α-glucosidase (37%). After colonic fermentation, a certain degree of inhibitory effects on α-amylase and α-glucosidase were present. In particular, throughout the entire *in vitro* GI digestion process, kiwifruit wine almost always exhibited the highest inhibition ratio for both α-amylase and α-glucosidase (except for α-glucosidase after colonic fermentation).

### Changes in forchlorfenuron in kiwifruit and kiwifruit products during in vitro GI digestion

**Fig. 7:**
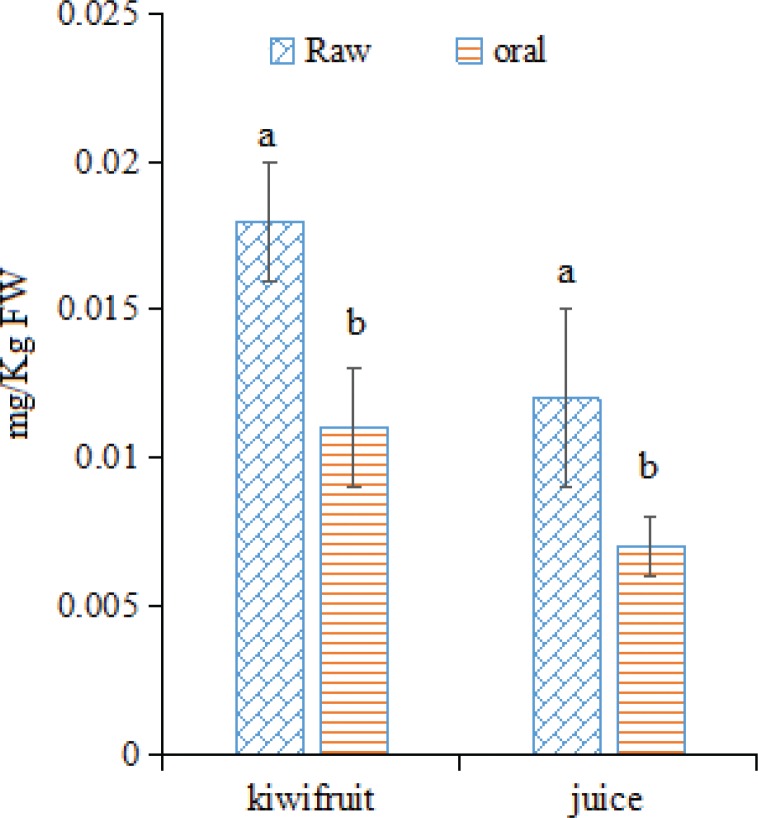
Changes in forchlorfenuron levels in kiwifruit and kiwifruit products during *in vitro* GI digestion. The different lowercase letters indicate significant differences among the different kiwifruit products with the same kiwifruit variety at *p* < 0.05.

Fruit-expanders, especially forchlorfenuron, have played an important role in increasing kiwifruit production (13). However, the long-term, excessive, and large-scale use of forchlorfenuron in the past has seriously eroded consumer confidence in Chinese kiwifruit, causing decreases in Chinese kiwifruit prices (3). Therefore, the levels of forchlorfenuron in kiwifruit were detected in this study (Fig. 7). Only 0.018 mg/Kg FW forchlorfenuron was observed in kiwifruit, which was much lower than the national standard of China (CNS GB 2763-2014, 0.05 mg/kg) (40). This result might be because under the strict management and effective promotion to farmers in recent years in China (3), the long-term, excessive, and large-scale use of forchlorfenuron has been greatly improved. In kiwifruit products, forchlorfenuron was detected only in juice (0.012 mg/kg FW forchlorfenuron). No forchlorfenuron was detected in kiwifruit wine, vinegar, dried slices, and jam, indicating that food processing such as fermentation and thermal treatment significantly influences the levels of forchlorfenuron in foodstuffs. After the *in vitro* GI digestion process, only trace amounts of forchlorfenuron were detected after the oral digestion process, whereas no forchlorfenuron was detected in samples after the other digestion processes, especially in the serum-available fractions, indicating that no forchlorfenuron would be absorbed in humans and that no harmful effects would be caused. However, special attention should be given to the possibility that forchlorfenuron may be catabolized into other metabolites (13), such as forchlorfenuron-4-O-β-D-glucoside, forchlorfenuron-3-O-β-D-glucoside, 4-hydroxyphenyl-forchlorfenuron, and 3-hydroxyphenyl-forchlorfenuron, which were not detected in the present study, and the impacts of which on human health are unclear.

### Effects of consuming kiwifruit and kiwifruit products on some selected intestinal microbiota

**Fig. 8:**
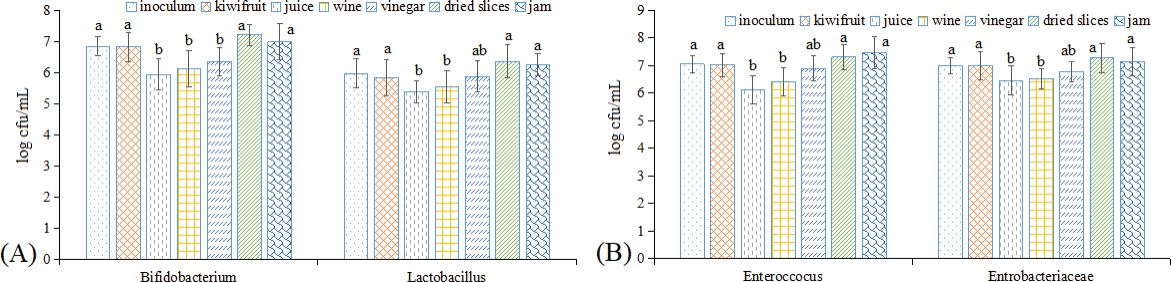
Changes in the abundances of some selected intestinal microbiota after consuming kiwifruit and kiwifruit products under *in vitro* GI digestion. (A) *Bifidobacterium* and *Lactobacillus* and (B) *Enterococcus* and *Enterobacteriaceae*. The different lowercase letters indicate significant differences among the different kiwifruit products with the same kiwifruit variety at *p* < 0.05.

After stomach digestion, foodstuffs are transported into the small intestine, and after nutritional substances are absorbed into the serum, the remaining material is transferred to the colon, which contains a diverse ecosystem of microorganisms. Nutritional substances can serve as substrates for the colonic microorganism community and influence the microorganism ecosystem (31, 33, 41, 42). Thus, we investigated changes in four important intestinal microbes, namely, *Bifidobacterium*, *Lactobacillus*, *Enterococcus*, and *Enterobacteriaceae*, after the digestion of kiwifruit and kiwifruit products (Fig. 8). After the colonic digestion process, the abundances of the tested microbes were observed to be between 10^5^ and 10^7^ cfu/mL, indicating that the survival rates of all four groups of microbes were quite high. Raw kiwifruit, kiwifruit juice, wine, and vinegar showed lower microbial abundances than were present in the inoculum. This result may be due to the higher polyphenol contents they contained, as polyphenolic compounds can exhibit concentration-dependent bactericidal properties in the colon (43), which could also explain why kiwifruit wine showed higher abundances than that were observed for kiwifruit juice and vinegar, as the wine contained the highest levels of polyphenols, followed by vinegar and juice. Dried kiwifruit slices and kiwifruit jam showed higher microbial abundances than that were present in the inoculum, possibly due to these two foodstuffs being enriched in fiber, which could promote microbial proliferation (44), although the polyphenol content in these samples was low, which would result in lower bactericidal properties. Thus, kiwifruit showed similar microbe concentrations to the inoculum, as kiwifruit contains high levels of fiber and polyphenols. Specifically, the abundances of *Bifidobacterium*, *Enterobacteriaceae*, and *Enterococcus* were higher than *Lactobacillus*, similar to the observations reported by Gumienna et al. (31).

### The proportion of the RNI or AI supplied by consuming kiwifruit and kiwifruit products

Based on the dietary guidelines for Chinese residents (45), the supplied nutritional substance proportions obtained from consuming 100 g of kiwifruit or kiwifruit products based on the initial concentration of nutritional substance in raw foodstuffs and the nutritional substance absorption for the recommended nutrient intake (RNI) or adequate intake (AI) value were estimated (Table 1). As no recommended values for N and polyphenol were available in the dietary guidelines for Chinese residents, these nutritional substances were not estimated in this study. Kiwifruit and kiwifruit products were observed to be a good source of VC, K, Mg, and Se, especially VC and Se, as consuming 100 g of kiwifruit per day could offer 54.86% VC and 25.51% Se based on the raw initial concentration, and 30.29% VC and 11.35% Se based on the nutrient absorption. In addition, dried kiwifruit slices and kiwifruit jam are also good sources of P, Fe, and Ca. Consuming raw kiwifruit was observed to be able to supply the most VC based on both the initial concentration and nutrition absorption. Furthermore, raw kiwifruit provided more of the estimated nutritional substances than all kiwifruit products. Previous reports (17, 46) also reported that citrus fruits offer more nutritional substances than fresh juice to humans.

**Table 1:** The proportion of nutritional substances supplied by consuming 100 g of kiwifruit or its products with respect to the recommended nutrient intake (RNI) or adequate intake (AI) value

The RNI or AI value		VC	Mn	N	K	Mg	Ca	Fe		Zn		Se	Na	P
								Male	Female	Male	Female			
		100 mg/d	4.5 mg/d	---	2000 mg/d	330 mg/d	800 mg/d	12 mg/d	20 mg/d	12.5 mg/d	7.5 mg/d	60 μg/d	1500 mg/d	720 mg/d
The supplied proportion based on the initial concentration of nutritional substance in raw foodstuffs (%)	kiwifruit	54.86	1.27	---	16.39	14.47	3.61	4.81	2.89	1.18	1.97	25.51	1.43	7.98
	juice	34.82	0.50	---	10.61	6.20	2.65	2.91	1.75	0.79	1.31	14.60	1.22	4.73
	wine	43.33	0.85	---	14.91	8.32	2.89	3.67	2.20	0.88	1.47	21.48	1.34	6.04
	vinegar	39.87	0.76	---	13.94	7.20	2.87	3.40	2.04	0.82	1.37	16.35	1.33	5.41
	dried slices	10.98	2.43	---	30.87	23.90	6.31	7.49	4.50	1.68	2.79	48.05	2.49	14.22
	jam	38.28	1.94	---	25.14	20.23	5.57	6.36	3.82	1.43	2.39	39.68	2.03	12.41
The supplied proportion based on the nutritional substance absorption (%)	kiwifruit	30.29	0.41	---	10.24	8.52	1.79	1.40	0.84	0.73	1.22	11.35	---	---
	juice	20.37	0.17	---	7.38	4.27	1.26	1.57	0.94	0.54	0.89	10.57	---	---
	wine	22.38	0.29	---	9.92	4.22	1.61	1.74	1.05	0.61	1.01	14.83	---	---
	vinegar	19.77	0.32	---	10.62	3.28	1.25	1.57	0.94	0.53	0.88	10.72	---	---
	dried slices	5.09	0.65	---	15.12	11.66	1.84	1.78	1.07	0.53	0.88	17.62	---	---
	jam	18.83	0.50	---	16.47	11.94	3.18	2.07	1.24	0.56	0.94	16.47	---	---

Note: ---, not available.

From the above discussion, we surveyed the nutritional properties and biological activities of kiwifruit and kiwifruit products. However, all these results were based on *in vitro* GI digestion. Although there are an increasing number of studies using *in vitro* digestion models to investigate the GI behavior of foods (18–19), it is still a fact that the *in vitro* simulation method does not account for every step of GI digestion, and most importantly, it does not fully mimic the active transportation processes. The *in vitro* results in this study are a prediction and reference for human nutritional studies. Hence, future studies *in vivo* and in the clinic are needed.

## Conclusions

From the results of this study, raw kiwifruit, kiwifruit juice, wine, and vinegar were observed to be rich in VC and polyphenol and also exhibited high biological activities, while dried kiwifruit slices and kiwifruit jam showed high mineral element contents. The fermentation products wine and vinegar showed similar levels of nutritional substances and were higher than those observed in juice, indicating that fermentation helps in the dissolution of nutritional substances. The thermally processed products, including dried kiwifruit slices and kiwifruit jam, showed similar levels of nutritional substances. During oral digestion, VC and polyphenols showed similar absorption characteristics, while mineral elements showed different trends. A good release rate of all nutritional substances was observed during stomach digestion, while the release rate in the serum-available, colon-available, and post-colonic fractions decreased. Thus, consuming dried kiwifruit slices and kiwifruit jam could supply more mineral elements, while consuming raw kiwifruit could supply the most comprehensive amounts of nutritional substances. Furthermore, juice and wine showed the highest nutritional substance release rates, while dried kiwifruit slices showed the lowest release rate. The biological activities detected in raw foodstuffs were much higher than those detected after *in vitro* GI digestion. What is more is that raw kiwifruit and kiwifruit wine showed the highest biological activities, while dried kiwifruit slices showed the lowest highest biological activities.
